# Effects of Nordic Walking Combined with High-Protein Intake and Time-Restricted Eating on Muscle-Related Outcomes in Postmenopausal Women: A Three-Arm Randomized Controlled Trial

**DOI:** 10.3390/nu18152470

**Published:** 2026-07-29

**Authors:** Yong Yang, Ya Zhu, Zbigniew Ossowski

**Affiliations:** 1 School of Sports Training, Chengdu Sports University, Chengdu 641418, China; yong.yang@awf.gda.pl (Y.Y.); zhuya@cdsu.edu.cn (Y.Z.); 2 Department of Physical Culture, Gdansk University of Physical Education and Sport, 80-336 Gdansk, Poland

**Keywords:** Nordic walking, time-restricted eating, dietary protein, muscle health, postmenopausal women, randomized controlled trial

## Abstract

Background: Muscle health decline is a major concern in postmenopausal women; however, most interventions have focused on exercise as an isolated component. It remains unclear whether embedding exercise within a broader lifestyle framework enhances muscle-related outcomes. Methods: A total of 100 postmenopausal women took part in this randomized controlled trial and were randomly assigned to three groups: Nordic walking combined with high-protein intake and time-restricted eating (NW+HP+TRE, *n* = 36), Nordic walking combined with time-restricted eating (NW+TRE, *n* = 33), and a control group (*n* = 31). The intervention lasted 12 weeks. The primary outcome was handgrip strength, and the secondary outcomes included physical performance (6 m gait speed and five times chair-stand time) and lean mass-related measures. Adherence to all intervention components was monitored throughout the study. Results: During the intervention, four participants did not complete the post-intervention assessment, resulting in 96 participants being included in the final analysis. Handgrip strength improved in both intervention groups, with the largest increase observed in the NW+HP+TRE group. Physical performance outcomes showed a similar pattern. In contrast, the NW+TRE group showed less favorable changes in lean mass-related measures, whereas the control group showed minimal overall changes. Adherence to Nordic walking, time-restricted eating, and protein intake was high in the intervention groups. Conclusions: A Nordic walking-based integrated lifestyle intervention was associated with a more favorable overall pattern of change in muscle strength, physical performance, and lean mass-related outcomes in postmenopausal women. These findings should be interpreted as differences between intervention packages rather than as evidence for the isolated effect of any single lifestyle component. Further research is needed to examine the long-term sustainability of such interventions and to clarify the contribution of individual components.

## 1. Introduction

Postmenopausal women are particularly vulnerable to declines in muscle health owing to aging, estrogen deficiency, and lifestyle-related changes in physical activity and diet [1,2]. This decline is characterized by reductions in muscle mass, strength, and functional performance, increasing the risk of frailty, falls, and loss of independence in later life. Among these indicators, handgrip strength has emerged as a practical and sensitive marker of early functional decline and is widely used in contemporary assessments of sarcopenia [2,3,4].

Muscle health in postmenopausal women should not be viewed as the result of exercise alone. In real-life settings, adaptation occurs within a broader physiological and behavioral context shaped by nutrient availability, metabolic regulation, daily routine, and long-term adherence [5,6]. However, many intervention studies still examine exercise as an isolated factor, with limited attention to the lifestyle conditions in which it is performed [5,7]. Given that muscle decline is influenced by multiple interacting processes, single-component interventions may not fully capture the complexity of the problem or reflect real-world conditions. This highlights the need for a more integrated lifestyle approach [5,6,7].

Nordic walking (NW) is a suitable exercise anchor for such an approach. As a pole-assisted walking modality, NW engages both the upper and lower limbs, increases whole-body muscle activation, enhances postural control, and provides a moderate and sustainable cardiovascular stimulus with relatively low joint stress [8,9]. These features make it particularly appropriate for community-dwelling older women. Compared with conventional walking, NW introduces a broader neuromuscular stimulus through active pole propulsion and coordinated gait mechanics, although its role in preventing muscle decline in postmenopausal women remains insufficiently studied [9,10].

In addition to physical activity, nutritional adequacy and eating-time organization may influence the extent to which exercise is translated into functional adaptation. Adequate protein intake is essential for supporting muscle protein synthesis, particularly in older adults who may exhibit anabolic resistance [11,12,13]. In parallel, time-restricted eating (TRE), which confines daily food intake to a defined time window, has attracted increasing interest as a behavioral strategy that may improve metabolic regulation and reinforce circadian organization [14,15]. Regularized meal timing may influence daily fluctuations in insulin sensitivity, substrate utilization, and metabolic efficiency, all of which may be relevant to the physiological environment in which exercise adaptation occurs.

This issue may be particularly relevant in postmenopausal women. Menopause is associated not only with hormonal changes but also with alterations in body composition, metabolic flexibility, and daily behavioral patterns, all of which may contribute to a higher risk of sarcopenia and functional decline [6,13,16]. From this perspective, TRE is not necessarily important because it is inherently anabolic, but because it may help structure the temporal environment of eating and metabolism. When combined with regular exercise and adequate protein intake, such temporal organization may provide a more supportive context for muscle-related adaptation.

Accordingly, exercise, protein availability, and meal timing can be viewed as interacting components within a broader lifestyle framework rather than as isolated exposures. This perspective is especially relevant for postmenopausal women, whose muscle health is often affected by insufficient protein intake, irregular daily routines, and reduced participation in structured physical activity [6,13,16]. A model that combines a feasible exercise modality with nutritional support and eating-time structure may therefore be more effective than exercise delivered under less supportive lifestyle conditions.

Therefore, the present study aimed to examine whether embedding exercise within an integrated lifestyle framework produces greater benefits for muscle-related outcomes than exercise delivered under less structured conditions. Using a three-arm randomized controlled design, we compared Nordic walking combined with high-protein intake and time-restricted eating, Nordic walking combined with time-restricted eating, and a control condition. We hypothesized that Nordic walking would improve handgrip strength and that the addition of protein optimization within a structured eating-time framework would further enhance muscle-related outcomes.

## 2. Methods

### 2.1. Study Design

This 12-week, three-arm randomized controlled trial was designed to evaluate the effects of a Nordic walking-based integrated lifestyle intervention on muscle health in postmenopausal women. Rather than treating exercise as an isolated exposure, the trial was structured to examine muscle-related outcomes within a broader lifestyle framework in which physical activity, nutritional support, and eating-time organization were combined in different configurations. Participants were randomly assigned to one of three groups according to a planned allocation ratio of 1:1:1:

(1) Nordic walking combined with high-protein intake and time-restricted eating (NW+HP+TRE, *n* = 36);
(2) Nordic walking combined with time-restricted eating (NW+TRE, *n* = 33);
(3) a control group receiving usual lifestyle guidance without structured exercise and dietary prescriptions (Control, *n* = 31).

The study was conducted under free-living conditions to reflect the practical realities of community-based lifestyle interventions.

All participants completed baseline assessments before randomization and were reassessed after the 12-week intervention period. The protocol was approved by the Ethics Committee of Chengdu Sport University (Approval No. Cheng Ti Lun Li (2025) 97) and was conducted in accordance with the Declaration of Helsinki. Written informed consent was obtained from all participants prior to enrolment.

The trial was registered with the Chinese ClinicalTrialRegistry (ChiCTR2500105425, 3 July 2025).

### 2.2. Intervention Framework

The intervention was conceptualized as an integrated lifestyle model centered around Nordic walking as the exercise anchor, with protein optimization and eating-window regulation incorporated as contextual modifiers of adaptation. In this framework, Nordic walking was intended to provide the primary mechanical and neuromuscular stimulus, while high-protein intake was used to support substrate availability for muscle adaptation, and time-restricted eating was implemented to structure daily eating behavior within a consistent temporal pattern. Accordingly, the three-arm design was used not only to compare intervention efficacy but also to explore whether embedding exercise within a more supportive lifestyle structure would be associated with more favorable muscle-related outcomes than exercise delivered with fewer accompanying lifestyle components.

### 2.3. Participants

Postmenopausal women were recruited from the community through health promotion activities, public outreach, and local advertisements. Menopausal status was defined as the absence of menstruation for ≥12 consecutive months. The target population was selected because postmenopausal women are at increased risk of muscle decline due to the combined influences of aging, hormonal changes, and lifestyle-related alterations in physical activity and dietary behavior.

Eligible participants were women aged 56–80 years who lived independently in the community, were able to walk without assistive devices, and were willing to comply with the intervention instructions. Participants were required to be free from contraindications to moderate-intensity exercise and capable of completing both baseline and post-intervention assessments.

The exclusion criteria included diagnosed cardiovascular, respiratory, neurological, or musculoskeletal conditions that could limit safe participation in exercise; severe metabolic or endocrine disorders; cognitive impairment that could interfere with compliance; current participation in structured exercise programs more than two times per week during the previous three months; and use of medications or treatments known to substantially affect muscle metabolism or body composition. Women with conditions that would prevent the safe implementation of the exercise or dietary protocol were also excluded.

All eligible participants received a full explanation of the study procedures and provided written informed consent prior to enrolment. Recruitment and screening were completed before random allocation.

According to the trial registration plan, the intended sample size was 90 participants, with 30 participants allocated to each group. During the actual recruitment process, 100 eligible postmenopausal women were ultimately randomized into the three study groups, resulting in a final sample that exceeded the originally planned target.

The age range of 56–80 years was selected to focus on community-dwelling postmenopausal women at relatively higher risk of age-related muscle decline while still being able to safely participate in a supervised walking-based intervention after screening.

### 2.4. Randomization, Allocation Concealment, and Blinding

After completion of the baseline assessments and confirmation of eligibility, participants were randomly allocated to one of the three study groups according to a planned allocation ratio of 1:1:1. The allocation sequence was generated by an independent statistician using a computer-generated randomization procedure in IBM SPSS Statistics for Windows, Version 28.0 (IBM Corp., Armonk, NY, USA). Although age stratification had been considered at the registration stage, the final allocation was performed without age-stratified assignment because the stratification scheme was not considered suitable during recruitment and allocation.

Allocation concealment was ensured using the sequentially numbered, opaque, sealed envelope (SNOSE) method. Each envelope contained one group assignment and was prepared, sealed, and stored by a research coordinator who was not involved in participant recruitment, baseline testing, intervention delivery, or outcome assessment. Following completion of baseline measurements, the next envelope in sequence was opened by designated personnel to determine group assignment. All allocation materials were archived for documentation and auditing purposes.

Owing to the nature of the intervention, participants and intervention staff could not be blinded to group allocation. However, the outcome assessors were not involved in the randomization process or intervention delivery and were assigned specific assessment tasks according to standardized procedures. In addition, after completion of the intervention period, all source data were de-identified prior to data entry and statistical analysis. Electronic datasets were managed using coded study IDs to minimize assessment and analytical bias.

### 2.5. Intervention Protocols

#### 2.5.1. Nordic Walking Training

Participants in the NW+HP+TRE and NW+TRE groups participated in a supervised Nordic walking program for 12 weeks. Training was conducted three times per week, yielding a total of 36 planned sessions, each lasting approximately 60 min. Each session included a standardized warm-up phase, a structured Nordic walking phase, and a cool-down period.

Each 60 min session consisted of approximately 10 min of warm-up, 40 min of Nordic walking, and 10 min of cool-down. The warm-up included low-intensity walking, joint mobilization, and dynamic stretching to prepare participants for the walking task. The main phase involved supervised Nordic walking performed within the prescribed heart-rate range, with continued reinforcement of proper pole use and coordinated arm–leg movement. The cool-down period included slow walking and light stretching exercises to facilitate recovery.

Exercise intensity was prescribed within a monitored target range and continuously monitored using wrist-worn heart rate monitors (Polar V800 GPS sport watch; Polar Electro Oy, Kempele, Finland) throughout each session. Participants were instructed to maintain exercise intensity within a monitored target range of approximately 60–75% of age-predicted maximal heart rate (HRmax = 220 − age), and not to exceed 75% HRmax. This range was selected to ensure a physiologically appropriate, safe, and sustainable training stimulus for community-dwelling postmenopausal women while providing sufficient cardiovascular and neuromuscular challenge. According to ACSM-based exercise intensity classifications, the selected range corresponds primarily to the upper portion of moderate intensity and approaches the lower threshold of vigorous intensity, and was therefore considered appropriate for the present supervised intervention context.

Standardized instruction in Nordic walking technique was provided before the intervention and reinforced throughout the training period. Participants were instructed in proper pole use, including coordinated arm–leg movement, active pole propulsion, trunk engagement, and symmetrical gait mechanics. Training progression was introduced gradually during the first two weeks (6 sessions) of the intervention until the target session structure and movement proficiency were achieved, after which the prescribed intensity range and session format were maintained for the remainder of the program.

All training sessions were supervised by trained staff to enhance safety, ensure technique fidelity, and improve intervention consistency.

#### 2.5.2. Time-Restricted Eating

Participants in both intervention groups were instructed to follow a time-restricted eating (TRE) protocol during the 12-week intervention period. Daily caloric intake was confined to a 10 h eating window, followed by a 14 h fasting interval. During the fasting period, participants were permitted to consume water and other non-caloric beverages.

Participants were encouraged to maintain a consistent daily eating schedule throughout the intervention period.

TRE was implemented as a behavioral timing strategy rather than as a weight-loss intervention. The purpose of including TRE in the present study was to provide a more structured daily eating rhythm and a potentially more regulated metabolic context in which exercise-related outcomes were evaluated. Standardized guidance on meal timing was provided at baseline and reinforced during follow-up.

#### 2.5.3. High-Protein Intake

In addition to Nordic walking and TRE, participants in the NW+HP+TRE group were instructed to achieve a daily protein intake target of approximately 1.4 g/kg/day throughout the intervention period. Nutritional counseling was provided at baseline and reinforced at regular intervals throughout the study. Guidance emphasized the consumption of high-quality protein sources distributed across meals, including dairy products, eggs, lean meat, fish, soy products, and legumes, according to participants’ habitual dietary patterns and feasibility of implementation. To improve data verification and dietary control, protein intake was monitored using self-reported food records (3-day meal plans and dietary records supported by the Mint Health app version 14.0.14) which were reviewed regularly by the research team. Daily protein intake was estimated using standard food composition tables, and participants received feedback when intake fell below the prescribed target. When necessary, dietary counseling was repeated to improve adherence to the protein prescription. Thus, achievement of the protein target was based not only on participant self-report, but also on systematic review and verification of dietary records by the study team.

The high-protein component was not treated as an isolated supplementation strategy but as a supportive nutritional condition intended to enhance the adaptive response to exercise. In this context, protein optimization functioned as a nutritional environment that facilitated muscle-related adaptation rather than as a standalone intervention.

The present study focused on protein-target attainment and adherence to the prescribed eating schedule rather than on a comprehensive comparative analysis of total energy intake or overall dietary composition across groups.

#### 2.5.4. Control Condition

Participants in the control group were instructed to maintain their usual daily routines throughout the 12-week study period and were not provided with structured Nordic walking training, time-restricted eating instructions, or protein-targeted nutritional guidance. To maintain participant contact and ethical balance, the control group received general health education materials but no individualized exercise or dietary interventions.

#### 2.5.5. Intervention Adherence and Fidelity

Intervention adherence was systematically monitored across all components, including exercise participation, time-restricted eating compliance, and protein intake. Attendance at Nordic walking sessions was recorded for each participant, and exercise adherence was calculated as the proportion of completed sessions relative to the total number of planned sessions (36 sessions over 12 weeks).

Compliance with the TRE protocol was assessed using self-reported daily logs and was defined as the proportion of days on which food intake was confined to the prescribed 10 h eating window relative to the total intervention period (84 days).

For the NW+HP+TRE group, protein intake adherence was evaluated as the proportion of days on which participants achieved the target intake of ≥1.4 g/kg/day relative to the total intervention period, based on reviewed dietary records.

As the study was conducted under free-living conditions, adherence was treated as a continuous measure rather than an all-or-none criterion. This approach allowed for a more realistic representation of intervention implementation and supported assessment of overall intervention fidelity.

### 2.6. Outcome Measures

Outcome assessments were conducted at baseline and after the 12-week intervention period by trained assessors following standardized procedures. All measurements were performed under consistent conditions to minimize variability.

#### 2.6.1. Primary Outcome: Handgrip Strength

Handgrip strength was assessed as the primary outcome measure using a calibrated handheld dynamometer (Jamar hydraulic hand dynamometer). Participants were tested in a seated position with the shoulder adducted and neutrally rotated, the elbow flexed at 90°, and the forearm in a neutral position. The wrist was maintained in slight extension according to standardized testing guidelines.

Although Nordic walking is not a conventional strength-training modality, its active pole propulsion, coordinated upper-limb use, and whole-body engagement support the use of handgrip strength as a clinically relevant muscle-related outcome in the present study.

Participants performed three maximal voluntary contractions with each hand, with adequate rest between trials to avoid fatigue. For each hand, the highest value across trials was recorded, and the mean of the left- and right-hand values was used for analysis as a representative measure of overall handgrip strength.

Handgrip strength is widely recognized as a practical and reliable indicator of overall muscle strength and is recommended as a core component of contemporary sarcopenia assessment frameworks.

#### 2.6.2. Secondary Outcomes: Physical Performance

Physical performance was evaluated using functional tests commonly applied in older adult populations.

The 6 m walk test was used to assess habitual gait speed. Participants were instructed to walk at their usual pace over a 6 m distance, and the time taken was recorded using a stopwatch. Gait speed (m/s) was calculated by dividing the distance by time.

Lower-extremity functional performance was assessed using the five-times chair stand test (FTCS). Participants were instructed to rise from a seated position to a full standing position and return to a sitting position five times as quickly as possible without using their arms. The total time required to complete five repetitions was recorded. A shorter completion time indicated better lower-limb function.

#### 2.6.3. Body Composition and Lean Mass-Related Outcomes

Body composition was assessed using bioelectrical impedance analysis (BCA-2A, Tsinghua Tongfang, Beijing, China). Participants were measured under standardized conditions, including fasting status and avoidance of strenuous physical activity prior to assessment. Appendicular lean mass (ALM) was calculated as the sum of the lean masses of the four limbs. The appendicular skeletal muscle index (ASMI) was derived by normalizing ALM to height squared (kg/m^2^). These indicators are commonly used to evaluate muscle mass status in older adults. In addition to the overall lean-mass indices, segmental lean masses of the upper and lower limbs were recorded, where applicable, to provide a more detailed description of body composition changes.

#### 2.6.4. Anthropometric Measures

Body weight and height were measured using standardized equipment, with participants wearing light clothing and no shoes. Body mass index (BMI) was calculated as body weight divided by height squared (kg/m^2^).

#### 2.6.5. Adherence-Related Measures

In addition to physiological outcomes, adherence-related measures were collected to evaluate intervention implementation. These included Nordic walking attendance rate, compliance with the TRE protocol, and achievement of the prescribed protein intake target, as described in Section 2.5.5.

### 2.7. Statistical Analysis

All statistical analyses were performed using R version 4.5.1 (R Foundation for Statistical Computing, Vienna, Austria). Continuous variables are presented as mean ± standard deviation (SD), and categorical variables are presented as frequencies and percentages where appropriate. Baseline characteristics were compared among the three groups using one-way analysis of variance (ANOVA) for continuous variables. For intervention outcomes, within-group changes were described from baseline to 12 weeks, and between-group differences in post-intervention values were evaluated using analysis of covariance (ANCOVA), with group entered as the fixed factor and the corresponding baseline value included as a covariate. Descriptive changes are presented as mean ± standard deviation (SD) to facilitate interpretation of the direction and magnitude of response. A two-sided *p* value < 0.05 was considered statistically significant.

## 3. Results

### 3.1. Participant Flow and Baseline Characteristics

**Figure 1:**
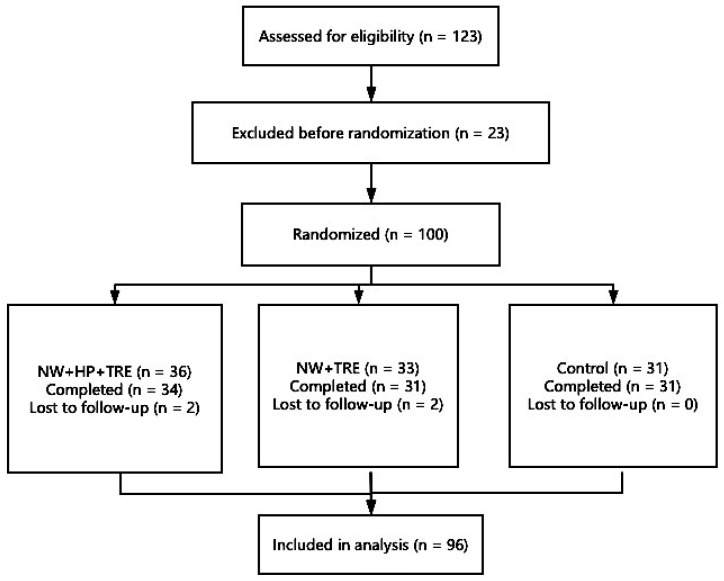
Flow diagram.

A total of 123 postmenopausal women were assessed for their eligibility. Of these, 23 did not proceed to randomization because of screening exclusion, withdrawal, or other reasons. The remaining 100 participants were randomized into three groups: NW+HP+TRE (*n* = 36), NW+TRE (*n* = 33), and control (*n* = 31) (Figure 1).

During the 12-week intervention period, four participants did not complete the post-intervention assessment (two in the NW+HP+TRE group and two in the NW+TRE group), whereas all participants in the control group completed the follow-up. Therefore, outcome data were available for 96 participants (NW+HP+TRE, *n* = 34; NW+TRE, *n* = 31; control, *n* = 31), corresponding to an overall retention rate of 96.0%.

The baseline characteristics are presented in Table 1. The three groups were comparable in terms of sample size and most baseline variables. The mean BMI values were 22.08 ± 2.47 kg/m^2^ in the NW+HP+TRE group, 22.95 ± 3.01 kg/m^2^ in the NW+TRE group, and 22.32 ± 2.02 kg/m^2^ in the control group. Baseline grip strength, chair stand performance, and body composition measurements were generally similar across the groups. Significant between-group baseline differences were observed in age, height, and 6 m gait speed. Therefore, between-group comparisons of intervention outcomes were performed using baseline-adjusted ANCOVA models.

### 3.2. Intervention Adherence and Fidelity

Adherence to the intervention protocol was high in both groups. Attendance at the supervised Nordic walking sessions averaged 95.45% in both the NW+HP+TRE and NW+TRE groups. Compliance with the time-restricted eating protocol was also high, with adherence rates of 94.11% and 94.81% in the NW+HP+TRE and NW+TRE groups, respectively.

In the NW+HP+TRE group, participants achieved the prescribed protein intake target (≥1.4 g/kg/day) on 94.05% of the intervention days. No crossover between the groups was observed.

### 3.3. Primary Outcome: Handgrip Strength

Handgrip strength improved in both intervention groups over the 12-week period, with the greatest increase observed in the NW+HP+TRE group. Changes in the primary and secondary outcomes across the groups are presented in Table 2.

The increase in overall grip strength (maximum value of either hand) was the largest in the NW+HP+TRE group, followed by the NW+TRE group, whereas no meaningful change was observed in the control group.

**Figure 2:**
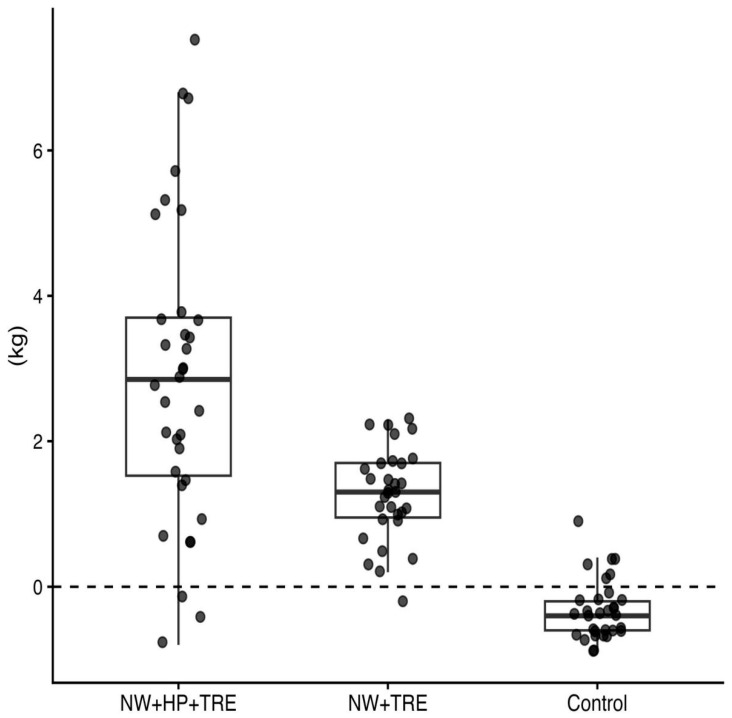
Changes in handgrip strength across groups.

When examined separately, left-hand grip strength increased by 4.06 ± 1.80 kg in the NW+HP+TRE group and by 0.83 ± 0.98 kg in the NW+TRE group, while it remained unchanged in the control group (−0.07 ± 0.55 kg). A similar pattern was observed for right-hand grip strength, with changes of +2.77 ± 2.01 kg, +1.36 ± 0.64 kg, and −0.25 ± 0.53 kg. The distribution of the changes in overall grip strength is shown in Figure 2.

To further address between-group comparisons, ANCOVA-adjusted pairwise differences with 95% confidence intervals were calculated for the primary and key secondary outcomes (Table 3). For handgrip strength, both intervention groups showed significantly higher adjusted post-intervention values than the control group, and the NW+HP+TRE group also remained significantly higher than the NW+TRE group. A similar pattern was observed for gait speed and chair-stand performance. For appendicular lean mass and ASMI, the NW+HP+TRE group showed significantly more favorable adjusted values than both the NW+TRE and control groups, whereas the difference between NW+TRE and control was not significant.

Adjusted between-group differences were estimated using ANCOVA, with post-intervention values as dependent variables, group as the fixed factor, and the corresponding baseline value as a covariate. Data are presented as adjusted mean differences with 95% confidence intervals. Negative values for chair-stand time indicate shorter completion time and therefore better performance.

### 3.4. Secondary Outcomes

#### 3.4.1. Physical Performance

Functional performance improved most prominently in the NW+HP+TRE group, followed by the NW+TRE group, whereas minimal changes were observed in the control group.

For 6 m walk time, the mean change was −0.39 ± 0.14 s in the NW+HP+TRE group, compared with −0.25 ± 0.16 s in the NW+TRE group and −0.01 ± 0.10 s in the control group. Correspondingly, the gait speed increased by 0.19 ± 0.07 m/s, 0.13 ± 0.08 m/s, and 0.01 ±0.06 m/s, respectively.

**Figure 3:**
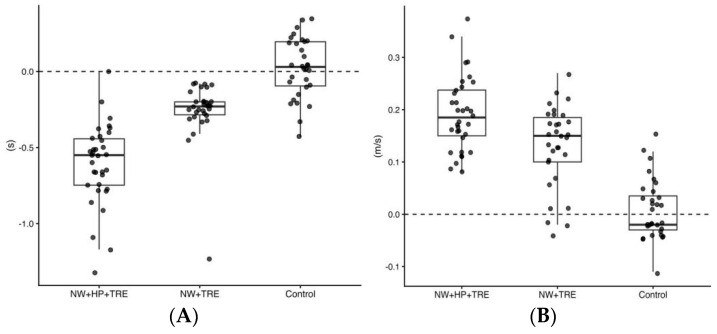
Changes in physical performance outcomes across the three groups after the 12-week intervention. (**A**) Chair-stand time. (**B**) Gait speed of 6 m.

For chair stand performance, the NW+HP+TRE group improved by −0.61 ± 0.27 s, compared with −0.26 ± 0.20 s in the NW+TRE group, while no change was observed in the control group (+0.02 ± 0.20 s). Changes in chair-stand time and 6 m gait speed across the three groups are shown in Figure 3.

#### 3.4.2. Lean Mass-Related Outcomes

The NW+HP+TRE group showed increases in bilateral lower limb lean mass, whereas the NW+TRE group showed slight reductions, and the control group remained stable.

The left lower-limb lean mass changed by +0.26 ± 0.15 kg in the NW+HP+TRE group, compared with −0.07 ± 0.12 kg in the NW+TRE group and −0.00 ± 0.06 kg in the control group. The right lower limb lean mass changed by +0.21 ± 0.09 kg, −0.10 ± 0.09 kg, and 0.01 ± 0.07 kg, respectively.

**Figure 4:**
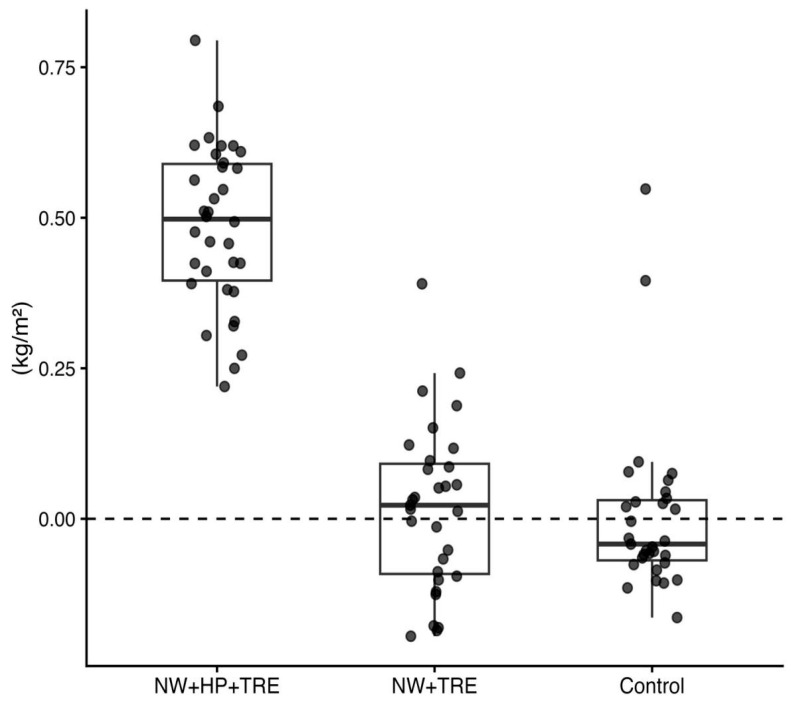
Changes in appendicular skeletal muscle index (ASMI) across the three groups after the 12-week intervention. The dashed horizontal line indicates no change from baseline (Δ = 0).

Total appendicular lean mass increased by +1.19 ± 0.30 kg in the NW+HP+TRE group compared with +0.04 ± 0.32 kg in the NW+TRE group and 0.00 ± 0.35 kg in the control group. Changes in ASMI across the three groups are shown in Figure 4.

#### 3.4.3. Body Weight and BMI

The NW+TRE group showed the largest reductions in body weight and BMI (−1.91 ± 2.03 kg and −0.81 ± 0.87 kg/m^2^, respectively), whereas the NW+HP+TRE group remained relatively stable (+0.19 ± 1.46 kg and +0.09 ± 0.59 kg/m^2^, respectively). The control group showed minimal change (+0.35 ± 0.99 kg and +0.14 ± 0.39 kg/m^2^).

### 3.5. Overall Pattern of Response

A consistent graded pattern was observed across the outcomes. The NW+HP+TRE group showed the most favorable improvements in muscle strength, physical performance, and lean mass-related measures. The NW+TRE group demonstrated moderate improvements, primarily in functional outcomes, whereas the control group showed minimal changes in functional outcomes.

## 4. Discussion

### 4.1. Principal Findings

This randomized controlled trial examined the effects of a Nordic walking-based integrated lifestyle intervention on muscle-related outcomes in postmenopausal women. The principal finding was that the group receiving Nordic walking combined with high-protein intake and time-restricted eating showed the most consistent favorable pattern of change across handgrip strength, physical performance, and lean mass-related outcomes.

Although both intervention groups demonstrated improvements in selected outcomes, the integrated lifestyle group showed greater gains in muscle strength and functional performance, along with more favorable changes in lower limb and appendicular lean mass [17,18]. In contrast, the group receiving Nordic walking combined with time-restricted eating but without protein optimization showed a less favorable body composition profile despite some functional improvement, whereas the control group remained largely stable over the 12-week period.

These findings suggest that muscle-related adaptations in postmenopausal women may depend not only on participation in exercise but also on the broader lifestyle context in which exercise is performed. In the present study, Nordic walking served as the shared exercise stimulus across the two intervention groups; however, the magnitude and quality of adaptation differed according to the accompanying nutritional and behavioral conditions. From this perspective, the added value of the NW+HP+TRE condition should not be interpreted simply as the result of “more components,” but rather as the effect of embedding exercise within a more supportive adaptive environment [5].

### 4.2. Interpretation Within an Integrated Lifestyle Framework

A central contribution of the present study is that it moves beyond the conventional logic of single-component exercise interventions. In many intervention studies, exercise is treated as an isolated exposure, as though its effects occur independently of the nutritional and behavioral contexts surrounding it. However, muscle adaptation in daily life rarely occurs under isolated conditions [18]. In postmenopausal women, whose muscle health is influenced by aging, estrogen deficiency, altered dietary patterns, and reduced physiological resilience, the response to exercise is likely shaped by multiple interacting factors.

Thus, the present findings support a more integrated interpretation of intervention design. Nordic walking can be understood as the primary mechanical and neuromuscular stimulus; protein optimization may provide a more favorable substrate environment for adaptation; and time-restricted eating may contribute to a more structured behavioral and metabolic context. These elements should not be viewed as merely additive components but as interrelated parts of a broader lifestyle framework within which adaptation takes place [9]. Accordingly, the results of the present trial are consistent with the possibility that muscle-related outcomes are influenced not only by whether exercise occurs but also by whether exercise is embedded within conditions that support its adaptive effects [8,19].

Importantly, this interpretation does not imply that all lifestyle components contribute equally or independently of each other. The present study was not designed to isolate the mechanistic effects of each component, and the relative contributions of exercise, protein optimization, and meal timing cannot be fully disentangled within the current three arm structure. Nevertheless, the observed graded pattern across groups supports the broader idea that lifestyle structure matters when exercise is used as a preventive strategy for muscle decline in postmenopausal women. The present findings should therefore be understood as comparisons between intervention packages delivered within different nutritional and behavioral contexts, rather than as evidence for the isolated mechanistic effect of any single component.

### 4.3. Nordic Walking as an Exercise Anchor

Nordic walking was selected as the exercise anchor for the intervention because it offers a form of whole-body physical activity that is feasible, scalable, and potentially more relevant to musculoskeletal function than ordinary walking alone. Unlike conventional walking, Nordic walking incorporates active upper limb propulsion, coordinated use of poles, trunk engagement, and greater postural involvement, thereby broadening the neuromuscular demands of locomotion. In postmenopausal women, this may be particularly relevant because an effective preventive exercise strategy should be safe, accessible, and acceptable, while still providing a sufficient stimulus to influence muscle-related outcomes. This broader relevance is also consistent with previous exercise-intervention studies in postmenopausal women showing that structured training programs can improve physical fitness and selected anthropometric or metabolic outcomes, even when the intervention model differs from the present Nordic walking-based approach [20,21].

The observed improvements in handgrip strength and functional performance in both intervention groups support the practical value of Nordic walking in this population [9,22,23]. Notably, these improvements were not limited to mobility-related measures. The direction of change in handgrip strength is consistent with the idea that pole-assisted walking may provide a broader training stimulus than lower-limb-dominant aerobic activity alone [24,25]. In this context, the use of handgrip strength as the primary outcome should be understood not as an attempt to evaluate Nordic walking as a pure strength-training modality, but as the assessment of a clinically relevant indicator of muscle function within a broader muscle-health framework. However, this interpretation should be approached with caution. The present study was not designed to directly compare Nordic walking with ordinary walking or resistance training, and no biomechanical, electromyographic, or metabolic measurements were collected to clarify the underlying mechanisms. Accordingly, the present findings support the usefulness of Nordic walking as a feasible exercise foundation, but they should not be considered as evidence that Nordic walking is inherently superior to other forms of exercise for all muscle-related outcomes.

A further practical advantage of Nordic walking is that it can be implemented in community settings with minimal equipment and relatively low technical barriers once basic instructions are provided. This is relevant for postmenopausal women, among whom long-term adherence may depend as much on feasibility and comfort as on physiological efficacy [26]. In this sense, the value of Nordic walking in the present study lies not only in its training stimulus but also in its suitability as a sustainable exercise platform within a broader lifestyle intervention.

### 4.4. The Role of Nutritional and Behavioral Context

One of the most important observations of the present study is that the addition of protein optimization was associated with a more favorable adaptation pattern than Nordic walking combined with time-restricted eating alone. This was evident not only in handgrip strength but also in lower limb and appendicular lean mass-related outcomes. These findings are biologically plausible. Adequate dietary protein is essential for supporting muscle protein synthesis [27,28] particularly in older adults, who may experience anabolic resistance and reduced adaptive remodeling efficiency. From this perspective, exercise provides the primary stimulus, but protein availability may influence the extent to which this stimulus is translated into tissue maintenance or gain [29,30].

Therefore, the present results suggest that the nutritional environment surrounding exercise is relevant. In the NW+HP+TRE group, participants were exposed to the same exercise regimen as those in the NW+TRE group but under conditions of more intentional protein support. This may help explain why the integrated intervention showed a more favorable lean-mass profile. However, the present findings should not be interpreted as demonstrating that protein alone was responsible for the between-group difference. A more cautious interpretation is that protein optimization may have enhanced the adaptive response to exercise within the specific behavioral structure used in the present study.

The NW+TRE group was also informative. On the one hand, participants in this group showed improved handgrip strength and several functional outcomes, indicating that Nordic walking combined with time-restricted eating was not ineffective. However, this group did not show the same favorable lean-mass response as the NW+HP+TRE group and, in some lower-limb lean-mass indicators, showed a slight decline. In addition, this group exhibited the greatest reductions in body weight and BMI.

This pattern requires careful interpretation. A reduction in body mass during lifestyle interventions is not inherently undesirable, particularly when accompanied by improved physical function. However, in the context of muscle health and sarcopenia prevention, weight reduction should not be automatically interpreted as a superior outcome. When viewed alongside the lower limb lean mass findings, the present results raise the possibility that time-restricted eating combined with exercise, in the absence of explicit protein optimization, may not provide the most supportive condition for preserving lean tissue in postmenopausal women.

This does not imply that time-restricted eating is inappropriate or harmful to this population. This suggests that its effects may depend on the nutritional conditions under which it is implemented. In the present trial, TRE should therefore be understood not as an independently validated anabolic strategy, but as a behavioral and temporal framework whose value may vary according to whether nutritional adequacy is maintained within the eating period. This interpretation is more consistent with the study design and avoids attributing causal effects to the TRE that cannot be isolated in the current intervention structure.

From a broader perspective, these findings have practical implications for community-based prevention strategies. Postmenopausal women often face a combination of declining muscle resilience, reduced habitual activity, and suboptimal dietary patterns; however, many are unlikely to adopt highly specialized or facility-dependent exercise programs. This broader relevance is also supported by recent evidence showing that physical activity levels in postmenopausal women are closely associated with abdominal obesity and metabolic markers, reinforcing the importance of lifestyle-oriented interventions in this population [30,31]. In this context, a Nordic walking-based integrated lifestyle intervention may represent a realistic preventive model because it combines accessibility with multidimensional support. The intervention can be implemented outside laboratory settings, requires limited equipment, and incorporates behaviors that can be integrated into daily life [8,31].

More broadly, the present findings support a shift in the conceptualization of muscle health interventions. Rather than asking which single exercise modality is effective, it may be more useful to ask under what lifestyle conditions an exercise stimulus becomes most beneficial. Such a perspective may be particularly relevant for healthy aging, in which physical activity, dietary patterns, and daily routines are closely interrelated in practice.

Therefore, the present study supports a more context-sensitive view of intervention design, especially for populations in which prevention must be feasible, acceptable, and sustainable.

### 4.5. Strengths and Limitations

This study had several strengths. First, the three-arm randomized controlled design enabled a comparison of different levels of lifestyle structuring around a shared exercise anchor, rather than simply contrasting one intervention with a passive control. This provides a more informative framework for examining how adaptation may differ across intervention contexts. Second, the intervention was implemented under free-living conditions with high adherence and retention, which enhanced the practical relevance of the findings. Third, the pattern of results was not limited to a single endpoint but extended across handgrip strength, functional performance, and lean mass-related outcomes, thereby supporting the interpretation of a broader muscle-related response.

This study has several limitations. The intervention duration was limited to 12 weeks, and a longer follow-up is needed to determine whether the observed changes are sustained over time. Some adherence measures, particularly dietary and eating-time compliance, relied on self-reports, which may have introduced reporting bias. The study population consisted exclusively of postmenopausal women, which improved population specificity but limited generalizability to other groups.

In addition, significant baseline between-group differences were observed in age, height, and gait speed. Although baseline-adjusted analyses were applied, residual imbalance cannot be fully excluded. A further limitation is that the study did not include a Nordic walking plus high-protein intake group without time-restricted eating. As a result, the present design cannot fully disentangle the independent contribution of protein optimization from the overall intervention package.

In addition, although dietary records were used to monitor adherence to protein-target attainment and eating-time prescriptions, the study did not include a comprehensive comparative analysis of total energy intake and overall nutrient composition across groups. Moreover, physical activity outside the structured exercise sessions was not systematically quantified. Therefore, the potential influence of non-intervention physical activity on the observed outcomes cannot be fully excluded.

In addition, minor imbalance in group size occurred after random allocation (36, 33, and 31 participants), which may arise in practice even under randomized assignment in free-living community-based trials. This does not invalidate the randomized design, but should be interpreted together with the baseline-adjusted analyses used in the present study.

Finally, the study was designed to compare lifestyle structures rather than isolate the mechanistic effects of each component. Accordingly, the respective contributions of Nordic walking, protein optimization, and time-restricted eating should be interpreted within an integrated intervention framework rather than as fully separable causal effects.

## 5. Conclusions

In this randomized controlled trial, a Nordic walking-based integrated lifestyle intervention was associated with a more favorable overall pattern of change in muscle-related outcomes in postmenopausal women than less structured conditions.

These findings are consistent with the view that muscle-related adaptation may depend not only on exercise participation but also on the broader lifestyle context in which physical activity is embedded. In the present study, combining Nordic walking with protein optimization and structured eating time patterns was associated with more coherent improvements in muscle strength, physical performance, and lean mass-related measures.

From a practical perspective, this integrated approach may represent a feasible strategy for supporting muscle health in community-dwelling, postmenopausal women. However, the present findings should be interpreted within the context of this study’s design. Future research is needed to examine the long-term sustainability of such interventions and further clarify the relative contributions of individual components within an integrated lifestyle framework.

## Figures and Tables

**Table 1:** Baseline characteristics of the participants.

Characteristic	NW+HP+TRE (*n* = 36)	NW+TRE (*n* = 33)	Control (*n* = 31)	*p* Value
Age, years	61.81 ± 7.00	65.67 ± 7.80	64.81 ± 4.81	0.027
Height, cm	157.96 ± 4.52	154.30 ± 5.11	158.45 ± 2.84	0.003
Body weight, kg	54.62 ± 8.16	54.58 ± 6.85	56.02 ± 5.67	0.635
BMI, kg/m^2^	22.08 ± 2.47	22.95 ± 3.01	22.30 ± 1.99	0.344
Overall grip strength, kg	25.63 ± 4.10	24.71 ± 5.42	25.75 ± 3.88	0.652
6 m gait speed, m/s	1.64 ± 0.18	1.77 ± 0.21	1.80 ± 0.17	0.002
5-times chair-stand time, s	6.75 ± 0.73	7.04 ± 1.70	7.25 ± 1.36	0.290
Appendicular lean mass, kg	15.87 ± 1.77	15.89 ± 1.32	16.04 ± 1.07	0.861
ASMI, kg/m^2^	6.47 ± 0.77	6.69 ± 0.65	6.39 ± 0.42	0.075

Data are presented as the mean ± SD. *p* values were derived from one-way ANOVA.

**Table 2:** Baseline, 12-week, and change values for primary and secondary outcomes across the three groups.

Outcome	NW+HP+TRE Baseline	NW+HP+TRE Week 12	NW+HP+TRE Change	NW+TRE Baseline	NW+TRE Week 12	NW+TRE Change	Control Baseline	Control Week 12	Control Change	*p* (Between-Group)
**Primary outcome**										
Grip strength, kg	24.39 ± 5.24	28.06 ± 5.25	+3.41 ± 1.91	23.52 ± 5.42	25.58 ± 3.94	+1.10 ± 0.83	24.27 ± 4.24	24.10 ± 4.18	−0.16 ± 0.54	<0.001
**Physical performance**										
6 m gait speed, m/s	1.64 ± 0.21	1.84 ± 0.21	+0.19 ± 0.07	1.77 ± 0.21	1.92 ± 0.16	+0.13 ± 0.08	1.80 ± 0.17	1.81 ± 0.17	+0.01 ± 0.06	<0.001
Chair-stand time, s	6.75 ± 0.73	6.08 ± 0.69	−0.61 ± 0.27	7.04 ± 1.70	6.49 ± 1.24	−0.26 ± 0.20	7.25 ± 1.36	7.28 ± 1.43	+0.02 ± 0.20	<0.001
**Lean mass-related outcomes**										
Lower-limb lean mass (left), kg	5.65 ± 0.40	5.94 ± 0.31	+0.26 ± 0.15	5.55 ± 0.34	5.52 ± 0.27	−0.07 ± 0.12	5.47 ± 0.32	5.47 ± 0.30	−0.00 ± 0.06	<0.001
Lower-limb lean mass (right), kg	5.78 ± 0.42	6.01 ± 0.39	+0.21 ± 0.09	5.61 ± 0.32	5.55 ± 0.33	−0.10 ± 0.09	5.63 ± 0.31	5.62 ± 0.29	−0.01 ± 0.07	<0.001
Appendicular lean mass, kg	15.92 ± 1.19	17.14 ± 1.03	+1.19 ± 0.30	15.89 ± 1.32	16.05 ± 1.11	+0.04 ± 0.32	16.04 ± 1.07	16.05 ± 1.02	+0.00 ± 0.35	<0.001
ASMI, kg/m^2^ *	6.50 ± 0.51	6.99 ± 0.51	+0.49 ± 0.13	6.73 ± 0.64	6.75 ± 0.58	+0.02 ± 0.14	6.39 ± 0.42	6.39 ± 0.43	+0.00 ± 0.14	<0.001
**Body composition**										
Body weight, kg	54.62 ± 7.87	54.80 ± 7.10	+0.19 ± 1.46	54.58 ± 6.85	52.67 ± 5.76	−1.91 ± 2.03	56.02 ± 5.67	56.37 ± 5.74	+0.35 ± 0.99	0.002
BMI, kg/m^2^	22.08 ± 2.47	22.17 ± 2.18	+0.09 ± 0.59	22.95 ± 3.01	22.14 ± 2.46	−0.81 ± 0.87	22.30 ± 1.99	22.44 ± 2.06	+0.14 ± 0.39	0.003

Footnote: Data are presented as mean ± SD at baseline and week 12, together with change values (week 12 − baseline). Between-group comparisons were evaluated using ANCOVA with baseline values entered as covariates. Sample sizes vary slightly across outcomes because some participants had missing 12-week values for specific measures. * ASMI = appendicular skeletal muscle index.

**Table 3:** ANCOVA-adjusted pairwise between-group differences with 95% confidence intervals for the primary and key secondary outcomes.

Outcome	Comparison	Adjusted Mean Difference	95% CI	*p* Value
Handgrip strength (kg)	NW+HP+TRE vs. NW+TRE	2.328	1.802 to 2.854	<0.001
	NW+HP+TRE vs. Control	3.590	3.063 to 4.116	<0.001
	NW+TRE vs. Control	1.262	0.724 to 1.800	<0.001
6 m gait speed (m/s)	NW+HP+TRE vs. NW+TRE	0.048	0.013 to 0.082	0.007
	NW+HP+TRE vs. Control	0.168	0.134 to 0.203	<0.001
	NW+TRE vs. Control	0.121	0.087 to 0.154	<0.001
Chair-stand time (s)	NW+HP+TRE vs. NW+TRE	−0.351	−0.462 to −0.239	<0.001
	NW+HP+TRE vs. Control	−0.640	−0.754 to −0.526	<0.001
	NW+TRE vs. Control	−0.289	−0.405 to −0.173	<0.001
Appendicular lean mass (kg)	NW+HP+TRE vs. NW+TRE	1.133	0.994 to 1.272	<0.001
	NW+HP+TRE vs. Control	1.170	1.031 to 1.309	<0.001
	NW+TRE vs. Control	0.036	−0.106 to 0.178	0.613
ASMI (kg/m^2^)	NW+HP+TRE vs. NW+TRE	0.450	0.384 to 0.517	<0.001
	NW+HP+TRE vs. Control	0.491	0.426 to 0.557	<0.001
	NW+TRE vs. Control	0.041	−0.028 to 0.110	0.240

## Data Availability

The data presented in this study are available from the corresponding author upon reasonable request. The data are not publicly available due to privacy and ethical restrictions.

## References

[1] Chen L.-K., Woo J., Assantachai P., Auyeung T.-W., Chou M.-Y., Iijima K., Jang H.C., Kang L., Kim M., Kim S. (2020). Asian Working Group for Sarcopenia: 2019 Consensus Update on Sarcopenia Diagnosis and Treatment. J. Am. Med. Dir. Assoc..

[2] Cruz-Jentoft A.J., Bahat G., Bauer J., Boirie Y., Bruyère O., Cederholm T., Cooper C., Landi F., Rolland Y., Sayer A.A. (2019). Sarcopenia: Revised European Consensus on Definition and Diagnosis. Age Ageing.

[3] Landi F., Liperoti R., Russo A., Giovannini S., Tosato M., Capoluongo E., Bernabei R., Onder G. (2012). Sarcopenia as a Risk Factor for Falls in Elderly Individuals: Results from the ilSIRENTE Study. Clin. Nutr..

[4] Adafer R., Messaadi W., Meddahi M., Patey A., Haderbache A., Bayen S., Messaadi N. (2020). Food Timing, Circadian Rhythm and Chrononutrition: A Systematic Review of Time-Restricted Eating’s Effects on Human Health. Nutrients.

[5] Beaudart C., Dawson A., Shaw S.C., Harvey N.C., Kanis J.A., Binkley N., Reginster J.Y., Chapurlat R., Chan D.C., Bruyère O. (2017). Nutrition and Physical Activity in the Prevention and Treatment of Sarcopenia: Systematic Review. Osteoporos. Int. J..

[6] Phillips S.M., Martinson W. (2019). Nutrient-Rich, High-Quality, Protein-Containing Dairy Foods in Combination with Exercise in Aging Persons to Mitigate Sarcopenia. Nutr. Rev..

[7] Liao C.-D., Liao Y.-H., Liou T.-H., Hsieh C.-Y., Kuo Y.-C., Chen H.-C. (2021). Effects of Protein-Rich Nutritional Composition Supplementation on Sarcopenia Indices and Physical Activity during Resistance Exercise Training in Older Women with Knee Osteoarthritis. Nutrients.

[8] Bullo V., Gobbo S., Vendramin B., Duregon F., Cugusi L., Di Blasio A., Bocalini D.S., Zaccaria M., Bergamin M., Nordic A. (2018). Walking Can Be Incorporated in the Exercise Prescription to Increase Aerobic Capacity, Strength, and Quality of Life for Elderly: A Systematic Review and Meta-Analysis. Rejuvenation Res..

[9] Tschentscher M., Niederseer D., Niebauer J. (2013). Health Benefits of Nordic Walking: A Systematic Review. Am. J. Prev. Med..

[10] Parkatti T., Perttunen J., Wacker P. (2012). Improvements in Functional Capacity From Nordic Walking: A Randomized Controlled Trial Among Older Adults. J. Aging Phys. Act..

[11] Morton R.W., Murphy K.T., McKellar S.R., Schoenfeld B.J., Henselmans M., Helms E., Aragon A.A., Devries M.C., Banfield L., Krieger J.W. (2018). A Systematic Review, Meta-Analysis and Meta-Regression of the Effect of Protein Supplementation on Resistance Training-Induced Gains in Muscle Mass and Strength in Healthy Adults. Br. J. Sports Med..

[12] Deutz N.E.P., Bauer J.M., Barazzoni R., Biolo G., Boirie Y., Bosy-Westphal A., Cederholm T., Cruz-Jentoft A., Krznariç Z., Nair K.S. (2014). Protein Intake and Exercise for Optimal Muscle Function with Aging: Recommendations from the ESPEN Expert Group. Clin. Nutr..

[13] Wolfe R.R., Cifelli A.M., Kostas G., Kim I.-Y. (2017). Optimizing Protein Intake in Adults: Interpretation and Application of the Recommended Dietary Allowance Compared with the Acceptable Macronutrient Distribution Range. Adv. Nutr..

[14] Regmi P., Heilbronn L.K. (2020). Time-Restricted Eating: Benefits, Mechanisms, and Challenges in Translation. iScience.

[15] Chaix A., Manoogian E.N., Melkani G.C., Panda S. (2019). Time-Restricted Eating to Prevent and Manage Chronic Metabolic Diseases. Annu. Rev. Nutr..

[16] Messier V., Rabasa-Lhoret R., Barbat-Artigas S., Elisha B., Karelis A.D., Aubertin-Leheudre M. (2011). Menopause and Sarcopenia: A Potential Role for Sex Hormones. Maturitas.

[17] Peterson M.D., Sen A., Gordon P.M. (2011). Influence of Resistance Exercise on Lean Body Mass in Aging Adults: A Meta-Analysis. Med. Sci. Sports Exerc..

[18] Liu C., Latham N.K. (2009). Progressive Resistance Strength Training for Improving Physical Function in Older Adults. Cochrane Database Syst. Rev..

[19] Phillips S.M. (2014). A Brief Review of Higher Dietary Protein Diets in Weight Loss: A Focus on Athletes. Sports Med..

[20] Church T.S., Earnest C.P., Morss G.M. (2002). Field Testing of Physiological Responses Associated with Nordic Walking. Res. Q. Exerc. Sport.

[21] Trabka B., Zubrzycki I.Z., Ossowski Z., Bojke O., Clarke A., Wiacek M., Latosik E. (2014). Effect of a MAST Exercise Program on Anthropometric Parameters, Physical Fitness, and Serum Lipid Levels in Obese Postmenopausal Women. J. Hum. Kinet..

[22] Shim J., Kwon H., Kim H., Kim B., Jung J. (2013). Comparison of the Effects of Walking with and without Nordic Pole on Upper Extremity and Lower Extremity Muscle Activation. J. Phys. Ther. Sci..

[23] Pellegrini B., Peyré-Tartaruga L.A., Zoppirolli C., Bortolan L., Bacchi E., Figard-Fabre H., Schena F. (2015). Exploring Muscle Activation during Nordic Walking: A Comparison between Conventional and Uphill Walking. PLoS ONE.

[24] Roy M., Grattard V., Dinet C., Soares A.V., Decavel P., Sagawa Y.J. (2020). Nordic Walking Influence on Biomechanical Parameters: A Systematic Review. Eur. J. Phys. Rehabil. Med..

[25] Kwak D.-J., Kim K.-T., Kang G.-M., Park Y.-J., Lee H.-L., Kim M.-K. (2019). Effect of 8-Week Nordic Walking Training on Nondominant Hand Grip and Shoulder Strength in Middle-Aged Women. J. Exerc. Rehabil..

[26] Nemoto Y., Sakurai R., Ogawa S., Maruo K., Fujiwara Y. (2021). Effects of an Unsupervised Nordic Walking Intervention on Cognitive and Physical Function among Older Women Engaging in Volunteer Activity. J. Exerc. Sci. Fit..

[27] Moore D.R., Robinson M.J., Fry J.L., Tang J.E., Glover E.I., Wilkinson S.B., Prior T., Tarnopolsky M.A., Phillips S.M. (2009). Ingested Protein Dose Response of Muscle and Albumin Protein Synthesis after Resistance Exercise in Young Men. Am. J. Clin. Nutr..

[28] Black K.E., Matkin-Hussey P. (2024). The Impact of Protein in Post-Menopausal Women on Muscle Mass and Strength: A Narrative Review. Physiologia.

[29] Tieland M., van de Rest O., Dirks M.L., van der Zwaluw N., Mensink M., van Loon L.J.C., de Groot L.C.P.G.M. (2012). Protein Supplementation Improves Physical Performance in Frail Elderly People: A Randomized, Double-Blind, Placebo-Controlled Trial. J. Am. Med. Dir. Assoc..

[30] Gregorio L., Brindisi J., Kleppinger A., Sullivan R., Mangano K.M., Bihuniak J.D., Kenny A.M., Kerstetter J.E., Insogn K.L. (2014). Adequate Dietary Protein Is Associated with Better Physical Performance among Post-Menopausal Women 60–90 Years. J. Nutr. Health Aging.

[31] Liu Y., Mao S., Xie W., Agnieszka H.-L.K., Helena S.M., Magdalena D.-Z., Qian G., Ossowski Z. (2024). Relationship between Physical Activity and Abdominal Obesity and Metabolic Markers in Postmenopausal Women. Sci. Rep..

